# Foundation models for cardiovascular diseases and medicine

**DOI:** 10.1093/ehjdh/ztag113

**Published:** 2026-07-16

**Authors:** Jiancheng Ye, Sophie Bronstein, Malak Abu Hashish

**Affiliations:** Weill Cornell Medicine, Cornell University, New York, NY, USA; Feinberg School of Medicine, Northwestern University, Chicago, IL, USA; Weill Cornell Medicine, Cornell University, New York, NY, USA; Touro University College of Osteopathic Medicine, Great Falls, MT, USA

**Keywords:** Foundation models, Cardiovascular disease, Artificial intelligence, Deep learning, Precision medicine, Digital health

## Abstract

Cardiovascular disease (CVD) remains the leading cause of mortality worldwide, accounting for more than 17 million deaths annually. Foundation models (FMs) are large-scale artificial intelligence systems pre-trained on broad datasets and increasingly studied for cardiovascular diagnosis, risk prediction, workflow support, and treatment personalization. This narrative review synthesizes evidence on FMs applied to cardiovascular medicine, including text-based, image-based, multimodal, and time-series models. The review examined model architectures, training strategies, performance metrics, clinical applications, implementation challenges, and evidence maturity using a three-tier framework. Cardiovascular FMs show promising but unevenly validated capabilities across multiple applications. Electrocardiogram (ECG)-based models such as ECGFounder have reported strong external validation performance for rhythm and conduction diagnoses, whereas imaging models such as Echo-Vision-FM support cardiac function assessment and pathology classification. Multimodal models, including CardioGPT and EchoCLIP, illustrate the potential value of integrating clinical text, imaging, and physiological signals, although several remain at a conceptual or proof-of-concept stage. Foundation models may become important tools in cardiovascular medicine by supporting earlier diagnosis, risk stratification, and clinical decision-making. However, the current evidence base remains heterogeneous, and major barriers persist, including limited interpretability, underrepresentation of diverse populations in training data, regulatory uncertainty, computational demands, and workflow integration challenges. Inclusive data development, transparent reporting, external validation, prospective clinical evaluation, and interdisciplinary implementation research are needed before these models can be deployed safely and equitably in routine cardiovascular care.

## Graphical Abstract

**Graphical Abstract:**
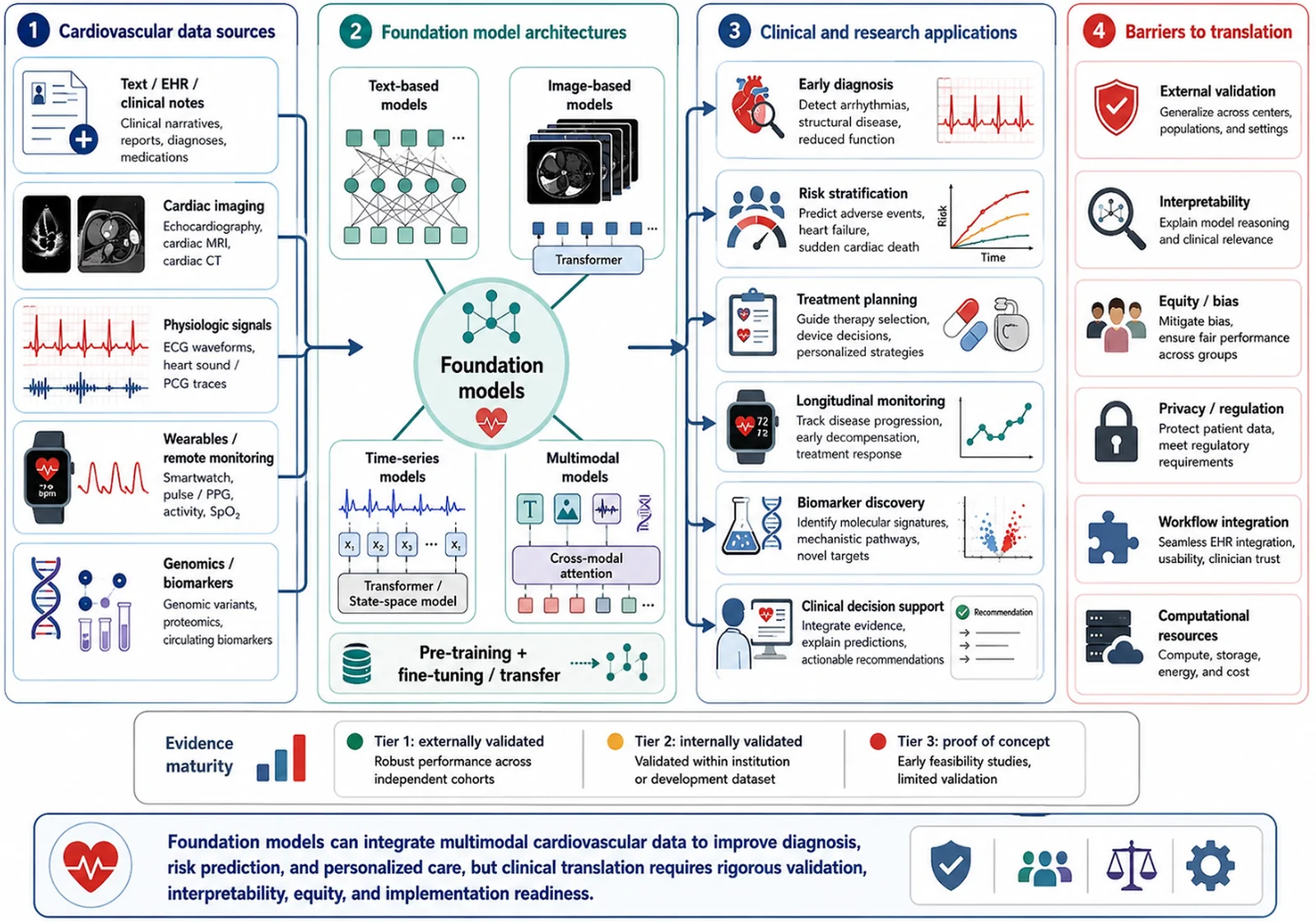

## Introduction

Cardiovascular disease (CVD) encompasses a diverse array of disorders affecting the heart and circulatory system, including coronary artery disease, arrhythmias, congenital heart defects, cardiomyopathies, and valvular heart diseases.^1^ Cardiovascular disease remains the leading cause of death globally, responsible for over 17 million deaths annually, with projections indicating an increase to approximately 23.6 million by 2030.^2^ Cardiovascular diseases account for nearly half of all non-communicable diseases, disproportionately affecting low- and middle-income countries (LMICs), where approximately 80% of cardiovascular-related deaths occur. In high-income countries such as the USA, CVD remains the leading cause of mortality across diverse demographic groups, with roughly one in five deaths attributed to this condition—totalling approximately 702 880 deaths in 2022 alone.^3^ The combined direct and indirect costs of heart disease, including healthcare services, medications, and lost productivity, amounted to an estimated $252.2 billion from 2019 to 2020.^4^ The economic impact is even more pronounced in LMICs, where CVDs are expected to account for nearly half of the $7.28 trillion in economic losses forecasted between 2011 and 2025.^2^

Despite significant advancements in medical technologies and clinical practices, substantial challenges persist in CVD diagnosis, treatment, and management. Coronary artery disease (CAD), predominantly caused by atherosclerosis, frequently remains asymptomatic until severe events such as myocardial infarction or stroke occur. In the USA, approximately 805 000 people experience heart attacks annually, with about 20% being ‘silent’ myocardial infarctions where patients remain unaware of cardiac damage until later stages, complicating timely intervention.^3^ Furthermore, disparities in cardiovascular healthcare delivery compound these challenges globally. Despite the availability of effective prevention programmes, persistent socioeconomic disparities lead to inequitable health outcomes. Populations in low- and middle-income countries face limited access to preventive healthcare services, increased exposure to cardiovascular risk factors, and reduced capacity to maintain health-promoting lifestyles, contributing to elevated cardiovascular morbidity and mortality in disadvantaged populations.^5^

Foundation models (FMs) represent large-scale pre-trained artificial intelligence (AI) systems developed through self-supervised learning on extensive, diverse datasets.^6^ These models learn generalized, semantically meaningful representations from vast amounts of unannotated data, often employing transformer architectures capable of modelling long-range dependencies in sequential and unstructured data through self-attention mechanisms.^7^ Unlike traditional machine learning (ML) approaches that require task-specific labelled datasets, FMs often use self-supervised, weakly supervised, or multimodal pretraining objectives that reduce reliance on task-specific labels. They can subsequently be fine-tuned or adapted to perform specific downstream tasks, providing versatility and flexibility across diverse applications.^8^ This generalizability may enable FMs to function effectively beyond their original training specifications without requiring substantial retraining or large-scale annotated datasets.^7^

The adoption of FMs in healthcare represents a significant departure from traditional ML approaches, which typically depend on task-specific labelled datasets and manual feature engineering. Well-known FMs such as Generative Pre-trained Transformer (GPT) and Bidirectional Encoder Representations from Transformers (BERT) have demonstrated remarkable adaptability and performance across various healthcare applications, including natural language processing (NLP), medical imaging interpretation, bioinformatics, and clinical decision support.^9^ Due to their extensive pre-training on vast, diverse datasets, FMs can capture generalized data representations that transcend individual tasks or narrow clinical applications.^10^ Growing research interest in healthcare FMs is largely attributed to their capacity for few-shot and zero-shot learning, significantly reducing the effort and costs associated with dataset construction and annotation.^7^

Foundation models represent a paradigm shift in ML, evolving from traditional techniques towards deep learning (DL) methodologies that emphasize broader adaptability, scalability, and efficiency.^10^ The capabilities enabled by FMs, including zero-shot and few-shot learning, dramatically expand their utility beyond the scenarios encountered during initial training.^8^ In diagnostic applications, FMs enhance the detection of subtle phenotypic variations and complex biological signatures, leading to improved diagnostic accuracy and enabling earlier disease identification.^11^ Additionally, multimodal FMs integrate information across different data types, including text, imaging, and biological datasets, to enable holistic and personalized patient care, holding significant promise for precision medicine.^12^ For example, AI-driven tools in cardiovascular medicine integrate multimodal data such as cardiac imaging, genomic markers, and wearable device metrics to support individualized risk stratification and treatment planning.^11^

Despite these advantages, the clinical implementation of FMs faces several obstacles related to reliability, accuracy, and ethical considerations. Potential vulnerabilities including hallucinations (generation of plausible but factually incorrect outputs), algorithmic biases, and inadvertent dissemination of misinformation should be thoroughly understood and mitigated before widespread clinical deployment.^7^ The complexity of FMs and their inherent ‘black-box’ nature present challenges for interpretability and validation, necessitating careful regulatory oversight and comprehensive ethical frameworks.^10^

This review aims to comprehensively examine the roles of FMs in CVD care and research, specifically focusing on how FMs are being adapted to support CVD diagnosis, risk prediction, treatment planning, and long-term disease management. We provide a detailed overview of current FMs applied to CVDs, including text-based, image-based, multimodal, and time-series models, and evaluate their performance, scalability, and trade-offs by analysing training datasets, architectural approaches, predictive accuracy, and real-world applications. Additionally, we identify critical knowledge gaps including the underrepresentation of diverse populations in training datasets, challenges in model transparency and explainability, and barriers to clinical integration and regulatory approval. By examining these factors, this review aims to inform future research directions, ethical deployment strategies, and interdisciplinary collaboration for the advancement of FMs in cardiovascular medicine.

## Methods

### Literature search strategy

We conducted a comprehensive literature search across multiple databases, including PubMed, EMBASE, CINAHL, Cochrane Library, IEEE Xplore, Web of Science, arXiv, and Google Scholar, for articles published or posted through June 2026.^13^ Search terms included combinations of ‘foundation models’, ‘large language models’, ‘transformers’, ‘deep learning’, ‘cardiovascular disease’, ‘cardiology’, ‘ECG’, ‘echocardiography’, ‘cardiac imaging’, and related terms. Eligible sources included peer-reviewed articles, preprints, and conference proceedings describing the development, validation, or application of FMs in cardiovascular medicine.

### Inclusion and exclusion criteria

Studies were included if they (i) described FMs or large-scale pre-trained models applied to cardiovascular conditions; (ii) reported on model architecture, training methodology, or clinical performance; and (iii) addressed diagnosis, risk prediction, treatment planning, or patient monitoring in CVD. We excluded studies focusing solely on traditional ML models without pre-training components, studies lacking sufficient technical detail, and those not related to cardiovascular applications.

### Review framing and evidence classification

This narrative review synthesizes a rapidly evolving and methodologically heterogeneous literature, spanning ECG, imaging, multimodal, and time-series FMs with differing architectures, sample sizes, and outcome metrics, into a clinically oriented framework.

Model maturity was assessed using a three-tier evidence-classification scheme applied to the models discussed in this review: Tier 1, externally validated—performance reported on at least one cohort, site, or population independent of the development data; Tier 2, single-cohort or internally validated—performance reported only within the development cohort (e.g. held-out internal test split) without independent external testing; and Tier 3, conceptual or proof-of-concept—architecture and intended use described, with simulated, preliminary, or no quantitative clinical performance reported. This scheme distinguishes models that are closer to clinical translation from models that remain exploratory.

### Data extraction and synthesis

From each included study, we extracted information on model architecture, training dataset characteristics, pre-training and fine-tuning methodologies, performance metrics, clinical applications, and reported limitations. We categorized FMs based on their primary data modality: text-based, image-based, time-series, and multimodal. We synthesized findings narratively, focusing on clinical applications, technical capabilities, and implementation challenges.^14^

## Overview of foundation models

Foundation models are large AI models pre-trained on massive volumes of general, task-agnostic datasets and subsequently adapted to a wide range of downstream applications through techniques including fine-tuning or prompt engineering.^8^ These models employ self-supervised learning on unannotated datasets, enabling them to learn detailed semantic representations that can be applied across multiple applications following fine-tuning with additional annotated data. Key characteristics include emergent capabilities such as zero-shot and few-shot prediction, which provide the ability to perform tasks without requiring significant additional training, and the capacity to handle multimodal datasets, making them versatile across different domains.^7^

Throughout this review, the term ‘foundation model’ refers to a model that (i) is pre-trained, typically via self-supervised or weakly supervised objectives, on a large and broad corpus, often multi-institutional or multi-task, rather than a single labelled task-specific dataset, and (ii) is designed for adaptation, via fine-tuning, prompting, linear probing, or zero-/few-shot transfer, to multiple downstream clinical tasks beyond the task used for pre-training. This definition distinguishes FMs from large but narrowly supervised DL systems trained end-to-end on a single labelled outcome, even when such systems are large, accurate, and clinically useful. Selected large supervised models, including CardioRiskNet, are presented as comparators and are explicitly distinguished from FMs meeting the study definition.

Some FMs have outperformed task-specific baselines on selected benchmarks by utilizing substantial computational power and extensive datasets to create a ‘foundation core’ that can be fine-tuned for specific applications. The pre-training process may include autoregressive language modelling or masked data prediction, allowing models to capture subtle patterns within data diversity without explicit labelling.^15^ For example, ChatGPT was trained using human-feedback-based alignment methods, which can improve instruction following, enhancing its ability to generalize across different applications.^16^

Examples of FMs include large language models (LLMs) such as BERT and GPT, which excel at NLP tasks, and contrastive language-image pre-training (CLIP), which aligns visual and textual representations for image-text tasks.^7^ ChatGPT has demonstrated remarkable capability in generating human-like text and performing tasks for which it was not explicitly trained.^8^ Bidirectional Encoder Representations from Transformers employs a bidirectional approach to encode sentences, excelling in NLP tasks, while GPT models focus on next-token prediction, enabling coherent text generation. Contrastive language-image pre-training bridges modalities by training on millions of image-text pairs, reflecting the multimodal capabilities inherent in FMs. Additionally, models like Stable Diffusion for text-to-image generation and Vision Transformers (ViT) for visual recognition demonstrate the extension of FMs into image synthesis and computer vision.^15^

In healthcare, particularly for CVD applications, FMs differ significantly from traditional methods such as logistic regression or convolutional neural networks (CNNs) in their pre-training and adaptation approaches.^7^ Conventional CVD models often require task-specific training from scratch using labelled datasets, whereas FMs leverage pre-trained representations that can be adapted to specific clinical tasks, reducing the need for large amounts of labelled data.^8^ Unlike logistic regression, which relies on predefined statistical assumptions, or CNNs, which require extensive annotated image datasets, FMs utilize large pre-trained knowledge bases that can be fine-tuned for CVD tasks with minimal additional data.^15^ Traditional models typically rely on predefined features or directly interpretable variables, limiting their capacity to detect complex and subtle relationships in multimodal medical datasets. In contrast, FMs leverage sophisticated DL architectures, including transformers, which effectively model complex dependencies and subtle signals across various data modalities.^10^ This capability is exemplified by their success in detecting electrocardiographic signatures of subclinical heart disease, enabling earlier detection and more precise prognostication compared to CNNs or logistic regression models.^11^ Foundation models also demonstrate superior generalization from large unlabelled datasets, effectively overcoming the limited transferability and narrow adaptability of traditional CVD models, which are typically trained for specific contexts.^10^ For example, ChatGPT can assist clinicians by summarizing reports or structuring clinical records—capabilities that standard models lack due to their narrow, task-oriented architecture.^16^ This paradigm shift enables FMs to better handle the complexity and variability of medical information through more efficient methodologies compared to traditional approaches.^15^

Foundation models in healthcare utilize diverse data types, including electronic health records (EHRs), imaging data such as electrocardiograms (ECGs) and magnetic resonance imaging (MRI), genomic data, and outputs from wearable sensors, enabling them to learn transferable representations across multiple modalities.^7^ The ability to leverage ECG signals, imaging reports, and data from wearables enables timely detection of conditions such as left ventricular systolic dysfunction, structural heart disease, and atrial fibrillation, which often manifest before symptoms develop or are detectable using conventional techniques.^17^ Furthermore, integration of genomic information facilitates precise identification of genetic markers associated with cardiovascular risk, enabling personalized therapeutic strategies and targeted interventions.^11^

The evolution from specialized, narrowly focused AI to generalized FMs represents a significant advancement in handling the complex characteristics of medical data.^7^ Specialized FMs like GatorTronGPT, trained on over 82 billion words from clinical text in EHRs, excel at tasks such as extracting information from unstructured notes.^18^ Imaging-based models such as MedSAM,^53^ trained on 1.5 million medical images including ECGs and MRIs, enable segmentation tasks relevant to CVD diagnosis. Additionally, data from wearable devices tracking heart rate or physical activity can be integrated into FMs, providing real-time monitoring capabilities, while genomic data enables models like Geneformer to map gene networks implicated in CVD risk factors.^15^ ChatGPT’s ability to process clinical narratives and summaries derived from wearable data further enhances its relevance in CVD contexts.^16^

Foundation models offer several advantages in healthcare and medical research, stemming from their scalability and generalizability. Pre-training on large datasets enables adaptation to diverse tasks with minimal additional training, significantly reducing dataset creation efforts. The scalability of FMs such as ChatGPT allows rapid digitization of clinical reports, reducing workload in CVD care.^16^ Moreover, FMs can be periodically updated or fine-tuned using new data, although such updates require careful revalidation to maintain robustness across populations and settings.^11^

However, these models face several limitations. Data privacy concerns arise from the use of sensitive patient information such as EHRs and genomic data, raising ethical and legal challenges. Another major challenge is interpretability; the black-box nature of FMs complicates understanding of decision-making processes in clinical settings where explainability is essential for trust and regulatory compliance. Foundation models can generate outputs based on learned probabilistic correlations without true semantic understanding, leading to medically incorrect outputs or ‘hallucinations’.^10^ Additionally, while FMs can accelerate AI advancement, they risk propagating biases or vulnerabilities across downstream applications—a significant concern in healthcare where fairness and safety are paramount.^8^ Therefore, careful clinical validation, thorough failure mode analysis, and rigorous testing of model outputs are necessary to ensure safety and reliability in healthcare applications.^11^

## Foundation models for cardiovascular disease

Foundation models applied to CVD diagnosis and treatment have emerged as versatile tools capable of processing large and diverse datasets to support diagnostic and prognostic tasks. *Table 1* categorizes FMs by data type and application domain, including text-based, image-based, multimodal, and time-series models. For each model, the table reports the model name, developers, year introduced, pre-training dataset characteristics, specific CVD applications, performance metrics, and clinical implications. Each model is also interpreted using the three-tier evidence framework defined in the Methods to clarify its current maturity and proximity to clinical translation.

**Table 1:** Categories of foundation models for cardiovascular disease

Model type	Model name	Developers	Year introduced	Pre-training dataset and scale	Specific CVD applications	Performance metrics	Implications
Text-based	BioBERT	Lee *et al*.^20^	2020	PubMed abstracts PMC full-text articles ∼18 billion words	Clinical note processing Risk factor extraction	Improved NER and relation extraction (F1 ∼0.85–0.90)	Supports downstream text-based ML models for CVD applications
	ClinicalBERT	Alsentzer *et al.*^21^	2019	MIMIC-III clinical notes ∼2 million notes	Diagnosis code prediction Readmission prediction	Improved AUC over baseline BERT (∼0.85)	Enhances structured insights from free-text EHRs
Image-based	Echo-Vision-FM	Zhang *et al.*^17^	2025	525 328 echocardiogram videos 4579 distinct patients	Cardiac function diagnosis and cardiac morphological value estimation	Accuracy 0.905, F1 0.941, AUC 0.931 for LVEF; AUC 0.849 MAE 3.87% and r^2^ 0.825 for LVEF; r^2^ 0.782/0.742 for end-systolic/diastolic volumes	Robust, generalizable echocardiogram analysis without manual labels, promising improved clinical diagnostics and utility in low-resource settings
	Vision FM for Cardiac MRI	Jacob *et al*.^22^	2024	36 million cardiac MRI images 27 000 patients	CMR segmentation Pathology classification Flow estimation	High segmentation accuracy Robust classification Generalizable cardiac MRI interpretation at scale	Supports general-purpose cardiac MRI workflows
	Transformer model for cardiac CT	Tölle *et al*.^23^	2024	Cardiac CTs from 8 hospitals 8104 CTs	Calcium scoring Valve anatomy Lesion assessment	Outperforms U-Net Better generalization	Scalable across hospitals with privacy preservation
	EchoFM	Kim *et al.*^24^	2024	20 million echo images 6500 patients	Cardiac chamber segmentation LVEF estimation Heart failure Valvular disease	State-of-the-art performance on evaluated echocardiography benchmarks >90% accuracy	Streamlines echo interpretation
Multimodal	EchoCLIP	Christensen *et al*.^25^	2024	1 million echo videos and cardiologist reports	Ejection fraction estimation Surgery/transplant tracking Zero-shot echo-to-text retrieval	Top-1 retrieval accuracy > 80% Strong multi-task alignment	Enables multimodal echo interpretation and clinical event tracking
	CardioRiskNet	Talaat *et al*.^26^	2024	Tabular and EHR data from real-world patient registry	Risk prediction Prognosis Feature attribution	AUROC up to 0.88 Precision-recall improvements	Improves risk stratification with interpretable AI
	ECG-PCG digital stethoscope model	Mathew *et al*.^27^	2024	PCG and ECG from clinical auscultations	Murmur and AF detection LV dysfunction	High task accuracy, AUROC > 0.90	Hybrid model mimics physician assessment
	CardioGPT	Fu *et al*.^28^	2024	Text, structured EHR, and ECG data	Risk scoring Medical Q&A Longitudinal summarization	Model simulations	Potential for LLM-based CVD care
	sEHR-ECG-Text	Lalam *et al*.^29^	2023	Structured EHR and ECG time series and text	Integrated diagnostics	Experimental results in fusion learning tasks	Blueprint for multimodal diagnostic models
Time-series	Apple Heart and Movement Study	Abbaspourazad *et al*.^30^	2024	141 K patients’ PPG/ECG over 3 yrs	Health status prediction Demographics Atrial fibrillation	Embedding correlation to clinical traits	Wearable-ready Population-level monitoring
	ECG-FM	McKeen *et al*.^31^	2024	2.5 million ECG segments	LVEF prediction Troponin abnormality Arrhythmias	Troponin AUROC: ∼0.91 LVEF AUROC: ∼0.92	Strong few-shot learning on ECGs
	Self-supervised transformer ECG model	Moody *et al*.^32^	2024	∼800 K unlabelled ECGs	Microvascular dysfunction Impaired LVEF	MFR AUROC: 0.76 LVEF AUROC: 0.95	Improved detection of subclinical CVD
	SleepFM	Thapa *et al*.^33^	2024	Sleep biosignals (EEG, ECG, PPG)	Sleep stage classification Circadian rhythm assessment	Strong classification performance in sleep staging tasks	Useful for monitoring sleep-related cardiac risks
	ECG-GPT	Khunte *et al*.^34^	2024	ECG waveform sequences	ECG generation Rhythm anomaly prediction	High sequence prediction accuracy	Could support generative ECG diagnostics and anomaly detection
	ECG-MAE	Hu *et al*.^35^	2023	Masked ECG segments	ECG feature learning Low-label classification	Improved label efficiency	Improves ECG classification with fewer annotations
	ECGFounder	Li *et al*.^36^	2024	10.7 million ECGs from HEEDB	150-label ECG classification Diagnoses arrhythmia, MI, LVEF, CKD, CHD, AF	AUROC > 0.95	High generalizability Works on both 12-lead and single-lead ECG
	Geneformer	Khan *et al*.^15^	2024	20 million gene expression profiles	Identification of CVD-linked genes	In early phases Not yet externally validated	Provides foundation for personalized cardio genomics and therapeutic discovery
	MedSAM	Ma *et al*.^37^	2024	1.5 million unlabelled medical images, including over 100 thousand cardiac MRI and echocardiographic images	Cardiac MRI segmentation and structural mapping	Outperforms supervised models in segmentation accuracy	Allows general cardiac image segmentation in low-label settings

Applying the evidence-classification scheme defined in the Methods, the models in *Table 1* distribute as follows: Tier 1 (externally validated): ECGFounder, Echo-Vision-FM, Vision FM for Cardiac MRI, the federated transformer for cardiac CT, EchoCLIP, ECG-FM, the self-supervised transformer model for myocardial flow reserve and LVEF, MedSAM, BioBERT, and ClinicalBERT; Tier 2 (single-cohort or internally validated): EchoFM, CardioRiskNet, the ECG-PCG digital stethoscope model, and the Apple Heart and Movement Study model; and Tier 3 (conceptual or proof-of-concept, lacking published external validation or formal benchmarking at the time of this review): CardioGPT, sEHR-ECG-Text, SleepFM, ECG-GPT, ECG-MAE, and Geneformer. This distinction is used throughout the review to prevent early-stage models from being interpreted as having the same evidentiary standing as externally validated systems.

### Text-based models

Text-based FMs are designed to interpret unstructured clinical documentation including physician notes, radiology reports, and discharge summaries. Natural language processing models such as BioBERT and ClinicalBERT have been evaluated for extracting entities and classifying biomedical or clinical text from large collections of EHRs and clinical notes by adapting to biomedical and clinical domains. BioBERT and ClinicalBERT excel at entity recognition and text classification in biomedical and clinical texts, respectively.^19^

### Image-based models

Image-based models specialize in analysing cardiac imaging data including echocardiograms, cardiac MRI, and CT scans. These models have been evaluated for detecting structural and functional cardiac abnormalities.

Echo-Vision-FM represents a significant advancement as a self-supervised video FM that employs masked auto-encoding on large unlabelled echocardiogram datasets to learn robust spatiotemporal features without manual annotations. The model enhances learned representations with a Spatial-Temporal Fusion Network (STF-Net), improving downstream performance on diverse clinical tasks including cardiac function diagnosis and morphological estimation. Echo-Vision-FM achieves state-of-the-art accuracy and generalizability across multiple datasets, outperforming existing supervised and self-supervised models for key cardiac metrics.^17^

Similarly, Vision FMs for Cardiac MRI, trained on 36 million MRI images, have been fine-tuned on 11 downstream tasks ranging from cardiac chamber segmentation to pathology classification, demonstrating high performance across multiple institutions and imaging protocols, thereby establishing their value as general-purpose MRI models.^22^ A complementary approach is represented by a federated transformer model for cardiac CT imaging, trained across eight German hospitals. This model leveraged knowledge from CNNs and demonstrated superior generalizability compared to traditional architectures, even when label availability was inconsistent across sites.^23^

### Multimodal models

Multimodal models incorporate multiple data types, primarily combining physiological signals to improve diagnostic accuracy. The ECG-PCG model records phonocardiogram (PCG) and ECG signals simultaneously using digital stethoscopes. By modelling temporal relationships between ECG and PCG, this approach leverages temporal and physiological correlations between cardiac sounds and electrical signals, providing significantly improved precision in detecting heart murmurs and atrial fibrillation.^27^

EchoCLIP represents another important multimodal FM that combines vision and language for echocardiogram interpretation. It integrates cardiac ultrasound videos with expert text reports, enabling tasks such as zero-shot retrieval, disease classification, and detection of clinical transitions including post-transplant states or surgical interventions. The model may support echocardiographic interpretation, retrieval, and downstream clinical decision support, particularly in data-limited contexts.^25^

Furthermore, CardioRiskNet is a hybrid model incorporating tabular EHR data with attention-based DL and explainable AI. In its source publication, CardioRiskNet is trained end-to-end in a supervised manner on a single registry-derived dataset rather than pre-trained via self-supervised learning on a broad, task-agnostic corpus; it is therefore best characterized as a hybrid, explainable supervised deep-learning model rather than a FM in the strict sense defined earlier. It is included here for comparative context because its transparent design supports cardiovascular risk prediction and prognosis while highlighting influential risk features.^26^

Several early-stage models are exploring approaches to unify diverse cardiovascular data streams into integrated diagnostic frameworks. sEHR-ECG-Text is a multimodal model combining structured EHR data, ECG waveforms, and clinical text. Its architecture includes separate encoders for each modality, fused using attention-based mechanisms to enable integrated cardiac risk assessment.^29^ Similarly, CardioGPT integrates LLMs with clinical signals and structured data. Its intended use cases include clinical question-answering, risk prediction, and personalized health summarization.^28^

### Time-series models

Time-series models focus specifically on continuous data streams from wearable sensors or long-term monitoring equipment. Transformer-based architectures are increasingly employed for their ability to model long-range temporal dependencies in biosignal data including ECGs, photoplethysmograms (PPGs), and other wearable sensor signals. Unlike traditional recurrent neural networks (RNNs) or long short-term memory networks, transformers leverage self-attention mechanisms, enabling them to capture complex temporal patterns more efficiently and with greater scalability.

One notable FM for wearable biosignals is trained using Apple Watch biosignal data collected in the Apple Heart and Movement Study (AHMS). Trained on PPG and ECG data from over 140 000 participants across 3 years, this model monitors wellness and aids in preventing severe medical conditions. The learned representations were associated with selected demographic characteristics and health conditions, demonstrating strong generalization across populations and potential for real-world monitoring applications.^30^

ECGFounder, another major time-series model, utilizes over 10.7 million ECG recordings and outperformed practicing cardiologists in F1 score across 20 conditions on an external, multinational test set, although the margin of advantage over cardiologists varied by diagnostic category, with the largest gains for conduction abnormalities and smaller gains for morphologically subtle findings. The model demonstrated strong performance even when adapted to single-lead ECG inputs, making it particularly valuable for mobile and wearable health applications. ECGFounder’s scale, accuracy, and cross-institutional generalizability establish a new benchmark for ECG-based diagnostics.^36^

Other models, while not specifically wearable-based, demonstrate how transformer-based pre-training on massive time-series datasets enables downstream flexibility. ECG-FM was trained on over 2.5 million ECG segments using contrastive learning and continuous signal masking. Following pre-training, the model was fine-tuned for tasks including detecting reduced left ventricular ejection fraction (LVEF) and abnormal troponin levels, showing strong performance with minimal labelled data.^31^ A similar approach was employed in an FM for ECG-based assessments of cardiac and coronary function, which utilized a self-supervised transformer model pre-trained on over 800 000 unlabelled ECG waveforms. The model was fine-tuned to identify impaired myocardial flow reserve (MFR) and reduced LVEF. Notably, the MFR predictor achieved superior performance and demonstrated significant associations with mortality risk across three test cohorts, highlighting the model’s value for both diagnosis and prognosis.^32^

Beyond these models, several emerging time-series FMs are gaining attention in cardiovascular AI. Models including SleepFM, ECG-GPT, and ECG-MAE have been proposed to address specialized tasks in biosignal analysis. SleepFM is designed to infer sleep stages from long-duration biosignal recordings, while ECG-GPT adapts transformer language modelling to predict ECG sequences, potentially useful for anomaly detection and rhythm forecasting. ECG-MAE employs a masked auto-encoder architecture to learn latent representations from partially occluded ECG waveforms, showing promise in low-label settings. Although these models remain under active development and are not yet widely deployed, they reflect the field’s growing emphasis on self-supervised and generative learning frameworks to scale ECG interpretation with minimal supervision.^19^

Beyond the unimodal time-series models above, a fast-growing line of work fuses ECG signals with natural language supervision to enable zero-shot diagnosis and language-grounded interpretation. HuBERT-ECG is a self-supervised ECG transformer pre-trained on approximately 9.1 million recordings spanning 164 conditions and has shown strong generalization across multiple independent external datasets, making it one of the most extensively validated ECG FMs to date.^38^ Several ECG–language contrastive and generative frameworks extend this paradigm, including MERL, which aligns ECG signals with paired clinical reports to enable zero-shot ECG classification^39^; C-MELT, a contrastive multimodal ECG–language pre-training framework^40^; D-BETA, which targets distribution shift between ECG and report embeddings; and ZETA, which couples zero-shot ECG diagnosis with interpretable, language-based reasoning, directly addressing the interpretability gap emphasized elsewhere in this review.^41^ Related question-answering systems, including an ECG language question-answering model, extend this paradigm towards few-shot clinical reasoning over ECG findings.^42^ Other recent studies have introduced related ECG-language and representation-learning architectures, including ECG-LM,^43^ GEM,^44^ HeartLang,^45^ and MEIT.^46^ In parallel, FMs pre-trained on photoplethysmography (PPG) signals from consumer wearables are emerging rapidly as a complement to ECG-based approaches, leveraging the much larger volumes of continuously collected PPG data available from smartwatches and fitness trackers to support arrhythmia screening, blood-pressure estimation, and general cardiovascular risk monitoring at population scale.

### Architectural overview

**Figure 1:**
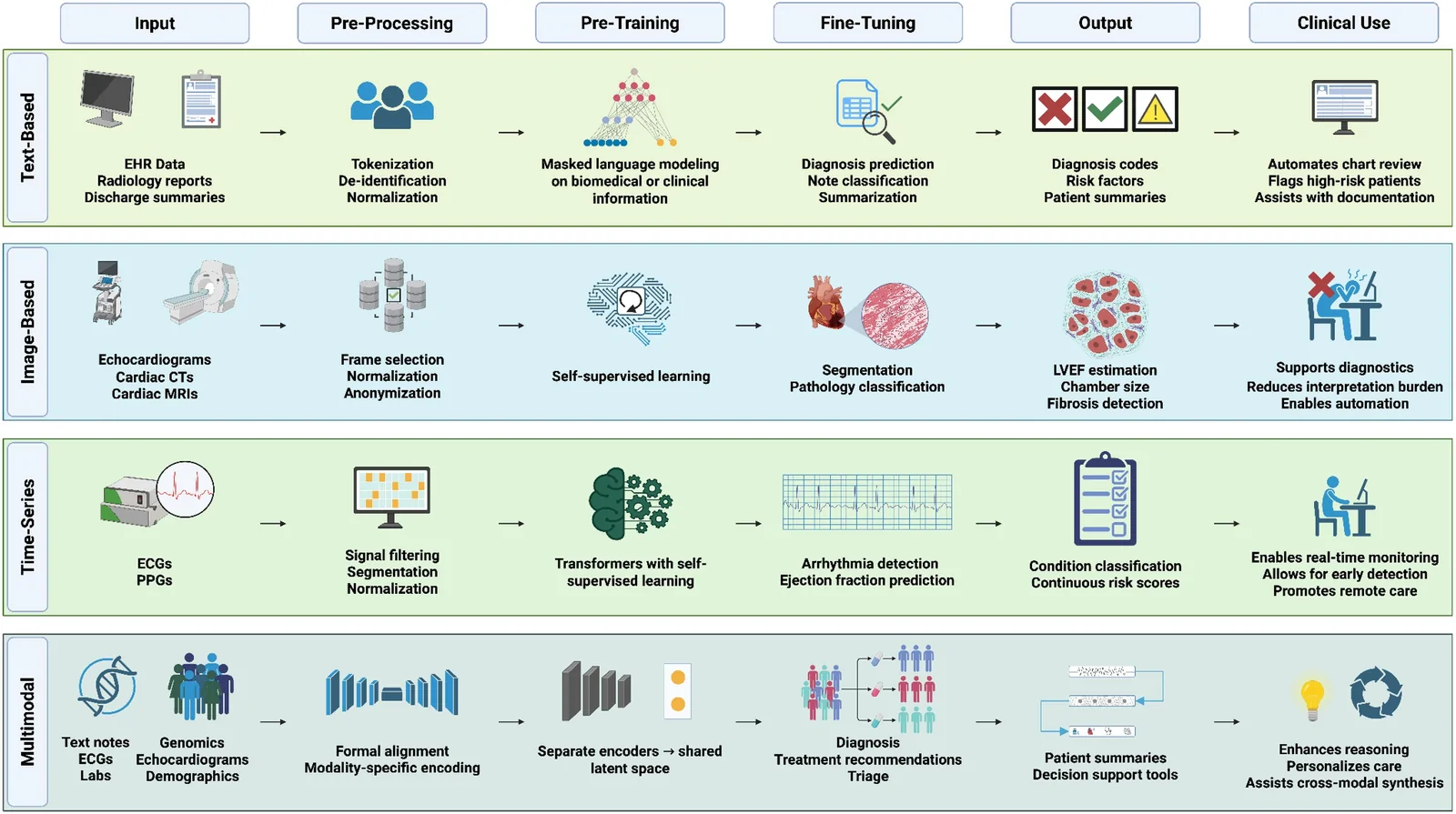
Overview of foundation model categories and their applications in cardiovascular care.

*Figure 1* illustrates the major categories of FM models used in cardiovascular care, including text, image, time-series, and multimodal models, and their differences in data processing, training methodologies, and clinical applications. Text-based models, built on LLM architectures, are trained on unstructured clinical text such as EHR notes and discharge summaries. They extract relationships, summarize documentation, and support clinical decision-making tasks including risk factor identification. Image-based models, incorporating CNNs and ViTs, are trained on medical imaging data including echocardiograms and CT scans. These models learn to detect anatomical and pathological features for disease detection and functional assessment. Time-series models, such as transformers and RNNs, process data from ECGs, telemetry, and wearable devices to capture temporal patterns and predict future cardiac outcomes. Multimodal models integrate diverse data types including text, images, signals, genomics, and environmental factors using fused architectures combining LLMs, CNNs, and transformers.^55^ These FMs enable comprehensive patient profiling and are applied in personalized diagnostics, prognosis, treatment planning, and digital twin-based clinical trial simulation.^56^

### Performance characteristics and trade-offs

*Table 2* summarizes the architectures, strengths, and limitations of CVD FMs. The comparison highlights architectural approaches, key strengths, and notable weaknesses across major cardiovascular FMs and related models. Transformer-based models such as EchoCLIP^25^ and Vision FM for Cardiac MRI^22^ demonstrate significant capabilities in imaging-related tasks, including segmentation and ejection fraction estimation, but require substantial computational resources.

**Table 2:** Architectural characteristics, strengths, and limitations of CVD foundation models

Model name	Architecture	Strengths	Weaknesses
BioBERT^20^	BERT Transformer	Biomedical-specific vocabulary understanding	Limited to formal biomedical language
ClinicalBERT^21^	BERT Transformer	Handles fragmented clinical narratives	Not generalized beyond EHRs
Echo-Vision-FM^17^	Self-supervised masked auto-encoder video encoder plus a Spatial-Temporal Fusion Network (STF-Net) that integrates spatiotemporal features for downstream tasks	State-of-the-art performance across multiple tasks and datasets, end-to-end video analysis, and reduced reliance on annotations	Dependence on video quality and distribution
Vision FM for Cardiac MRI^22^	SSL vision transformer	Large-scale, multi-task capable	MRI-specific and limited to image data High resource requirements for training
EchoFM^24^	Transformer with MAE and contrastive loss	Strong temporal and spatial modelling	Requires video-level echo data
Transformer model for cardiac CT^23^	Transformer and knowledge distillation	Works in federated and real-world settings	Complex training and label harmonization
EchoCLIP^25^	CLIP-style vision-language transformer	Cross-modal generalization Longitudinal tracking	High computational cost Large, paired dataset required
CardioRiskNet^26^	Hybrid attention-based deep neural network	Explainability Multimodal EHR compatibility	Limited generalizability without structured inputs
ECG-PCG digital stethoscope model^27^	Transformer and MAE	Models electrical and mechanical timing	Device-specific input format
CardioGPT^28^	LLM and multimodal encoders	Conversational Integrates varied clinical modalities	Still theoretical and not widely validated
sEHR-ECG-Text^29^	Fusion transformer with multimodal encoders	Handles full clinical picture Flexible design Enables reasoning over complete patient history Adaptable to various data sources	At an early stage, with uncertain clinical robustness Computationally intensive Complex real-world deployment
Apple Heart and Movement Study^30^	Transformer and contrastive SSL	Massive real-world data Scalable	Limited clinical task-specific benchmarks
ECG-FM^31^	Transformer and contrastive learning	Generalizable Open source	Requires curated ECG signals
Self-supervised transformer ECG model^32^	Vision transformer	SSL boosts performance with fewer labels	Performance may vary by cohort
SleepFM^33^	Transformer with temporal encoding	Specialized for long-duration biosignals	Limited cardiac-specific benchmarks
ECG-GPT^34^	GPT-style transformer	Predictive modelling Captures waveform dynamics	Still under experimental validation
ECG-MAE^35^	Masked auto-encoder	Strong representation learning with SSL	Requires tuning for different ECG tasks
ECGFounder^36^	RegNet	Extensive training Cross-domain generalization Compatible with single-lead and wearable ECG inputs	High computational requirements Explainability is still evolving
Geneformer^15^	Transformer encoder	Captures biological patterns from omics data Adaptable to molecular CVD profiling	Requires large, curated datasets Less interpretable to providers
MedSAM^37^	Vision transformer	Supports promptable segmentation across multiple medical-imaging datasets Generalizable across institutions	Lacks task-specific fine-tuning High computational resources needed for training

In contrast, time-series models such as ECG-FM,^31^ the AI-MFR Transformer,^32^ ECGFounder,^36^ and the Apple Heart and Movement FM^30^ may offer a more favourable performance-efficiency balance. These models can process short-duration ECG or PPG inputs, making them suitable for wearables and point-of-care applications. Although they may not capture the full anatomical complexity of image-based systems, their lower computational requirements and compatibility with real-time applications are important practical advantages.

Some models are designed for generalizability across data sources, clinical settings, and equipment types. The Federated Transformer for Cardiac CT,^23^ for example, performs consistently across sites due to diverse training data and privacy-preserving techniques such as federated learning. However, these models may demonstrate suboptimal performance for highly specialized clinical tasks without proper fine-tuning. This contrasts with domain-specific models such as CardioRiskNet^26^ or the Digital Stethoscope PCG + ECG FM,^27^ which are tailored to targeted applications—risk prediction and auscultation, respectively. While these models are computationally efficient and demonstrate excellent performance in specific domains, they tend to struggle when generalizing across larger or multimodal contexts.

As models increase in architectural complexity and data modality integration, interpretability often decreases. Multimodal systems such as CardioGPT and sEHR-ECG-Text^19^ show promise but are typically regarded as black-box models, making clinical trust and regulatory validation challenging. CardioRiskNet, in contrast, includes inherent explainability tools, enabling clinicians to visualize which features drive risk predictions. Although simpler in scope, CardioRiskNet’s transparency supports broader clinical adoption and safer deployment.^26^

Architectural choices in cardiovascular FMs reflect distinctive properties of cardiovascular data. Convolutional architectures, which dominated earlier ECG and echocardiography models, are effective for local morphological features such as QRS-complex shape or valve-leaflet edges, but they are less well suited to long-range periodicity and beat-to-beat variability that carry diagnostic information in arrhythmia and conduction system disease. This limitation helps explain the growing use of transformer-based architectures with self-attention in recent ECG and echocardiography video models. Masked auto-encoding objectives, used by Echo-Vision-FM, ECG-MAE, and EchoFM, are well matched to cardiovascular imaging and signals because cardiac structures and waveforms exhibit local redundancy across nearby frames or samples; in contrast, contrastive objectives used by EchoCLIP, ECG-FM, and MERL are most useful when paired text or multiview supervision is available and the goal is cross-modal alignment rather than dense reconstruction. Cardiovascular FM development is also constrained by heterogeneous acquisition hardware, including lead configurations, sampling rates, probe types, and frame rates, which can generate substantial covariate shift across institutions and countries. This challenge underscores the importance of multi-site, multi-device training strategies used by models such as the federated CT transformer and ECGFounder. Finally, the rarity and clinical heterogeneity of high-stakes endpoints, such as sudden cardiac death and inherited arrhythmia syndromes, limit the availability of labelled validation cohorts even when large pre-training corpora exist, making self-supervised and few-shot approaches especially relevant for cardiovascular AI.

Self-supervised learning models, including the AI-MFR Transformer, ECG-MAE, and ECG-GPT,^19^ can reduce reliance on labelled data by learning from large unlabelled datasets. These models may be especially useful for rare or poorly annotated CVD phenotypes and for resource-limited settings, although they still require carefully curated validation cohorts and structured clinical integration frameworks. Models such as EchoFM^24^ and the federated transformer for cardiac CT^23^ address real-world robustness by focusing on data heterogeneity, clinical variability, and privacy-preserving learning. Large-scale models such as the Apple Heart and Movement FM^30^ and ECGFounder^36^ demonstrate broad generalization potential, whereas specialized models such as the digital stethoscope PCG + ECG FM^27^ and EchoCLIP^25^ achieve strong performance in narrower domains but have more limited cross-task applicability.

## Applications in cardiovascular disease

### Enhanced diagnostic capabilities

Foundation models are being evaluated for CVD diagnosis by enabling earlier and more accurate detection of clinical abnormalities, frequently before symptom onset. Multimodal FMs combining synchronized ECG and PCG data have demonstrated improved detection accuracy for atrial fibrillation and heart murmurs compared to traditional rule-based methods.^27^ Transformer-based ECG models such as ECG-FM and ECGFounder have demonstrated strong performance in retrospective benchmark evaluations including hypertrophic cardiomyopathy and reduced ejection fraction with minimal labelled data, outperforming classical convolutional models in generalizability and operational efficiency.^36^

EchoFM, a vision transformer trained on echocardiographic sequences, performs segmentation and ejection fraction estimation with near-clinical accuracy, demonstrating potential for identifying structural disorders such as amyloidosis and pulmonary hypertension.^24^ Similarly, vision transformers trained on blood flow sound data can improve arteriovenous fistula stenosis detection—historically reliant on invasive methods—through identification of sound patterns undetectable by clinicians.^27^

### Risk stratification and prediction

Foundation models enable both personalized and population-level risk stratification by synthesizing multiple longitudinal datasets. Transformer-based models including CardioRiskNet, trained on EHR, have outperformed traditional risk calculators by capturing non-linear interactions between variables such as body mass index, age, and medication adherence.^26^ These models improve predictive accuracy while incorporating explainable AI elements to enhance clinical trust.

Additionally, FMs embedded in wearable devices, such as Apple Heart and Movement FM, enable continuous monitoring of cardiovascular risk factors. These technologies identify physiological aberrations associated with early risk indicators, such as reduced heart rate variability or altered movement patterns, may eventually support monitoring and risk-assessment applications; prospective evaluation of alerting or intervention workflows is needed.^30^ Integrating such time-series models into public health infrastructures could provide comprehensive prediction tools at the population level, enabling proactive resource allocation and prevention of adverse cardiovascular events.^32^

### Treatment planning and personalization

Foundation models may support the development of individualized cardiovascular care strategies. Large language models such as CardioGPT can synthesize patient history, medication records, and diagnostic results to provide context-specific treatment summaries, with the potential to support documentation or interpretation workflows.^19^ These models can derive insights from large-scale multimodal data to suggest personalized pharmacological or procedural interventions, essentially functioning as decision support assistants.

Furthermore, continuous integration with wearable data enables optimized therapeutic recommendations. Electrocardiogram-based FMs have been employed to evaluate treatment response in patients undergoing cardiac resynchronization therapy or recovering from myocardial infarction, providing insights into treatment efficacy and informing timely adjustments.^31^ Models such as CardioRiskNet can predict the magnitude of benefits from specific lifestyle modifications, including increased physical activity or reduced sodium intake, enabling physicians to tailor non-pharmacological interventions for optimal efficacy.^26^

### Research and biomarker discovery

Foundation models have contributed substantially to novel biomarker identification and drug target discovery. Geneformer, a genomics-informed FM, has been used to examine cardiovascular risk-related transcriptional profiles to identify gene clusters predicting susceptibility to heart failure or atherosclerosis.^15^ Additionally, unsupervised learning frameworks such as ECG-MAE and ECG-GPT enable researchers to explore latent disease phenotypes without prior assumptions. These models can reveal subgroups within established diagnostic categories—such as ischaemic vs. non-ischaemic heart failure based on waveform features—providing new avenues for drug development and clinical trials.^19^

### Preclinical and translational research

Foundation models are increasingly integrated into preclinical experimentation. FMs may complement *in silico* and preclinical research by learning representations from large experimental datasets; their effect on animal use and development timelines requires empirical evaluation.^22^ Through self-supervised learning, these models can infer missing data points or simulate pharmacological responses under varying conditions, serving as valuable tools for mechanistic insights. When integrated into comprehensive transformer models, preclinical datasets enable continual learning, improving generalizability for clinical applications. CMR-SSL, for example, was trained using unlabelled MRI data from preclinical studies and demonstrated strong performance across multiple institutions in downstream tasks including cardiac pathology classification and chamber segmentation.^19^

### Patient-centred and longitudinal care

Foundation models can facilitate a shift from disease-centred to person-centred cardiovascular care. By integrating into frameworks such as transitional care programmes, community follow-ups, and symptom self-reporting systems, FMs enable longitudinal tracking of patient trajectories. Models like CardioGPT and EchoCLIP can contextualize symptoms, helping distinguish benign variations from early signs of deterioration.^25^

In cardiac rehabilitation, FMs embedded in wearable or portable technologies can assess activity levels, treatment adherence, and physiological responses, enabling real-time personalization of post-discharge care plans. Additionally, community-based platforms integrating social determinants of health with clinical information can improve equitable distribution of follow-up care, particularly for marginalized populations.^30^ Ultimately, these person-centred models promote continuous and adaptive care systems in which AI supports clinical intuition without replacing it. Prospective studies are required to determine whether these approaches improve longitudinal cardiovascular outcomes.

**Figure 2:**
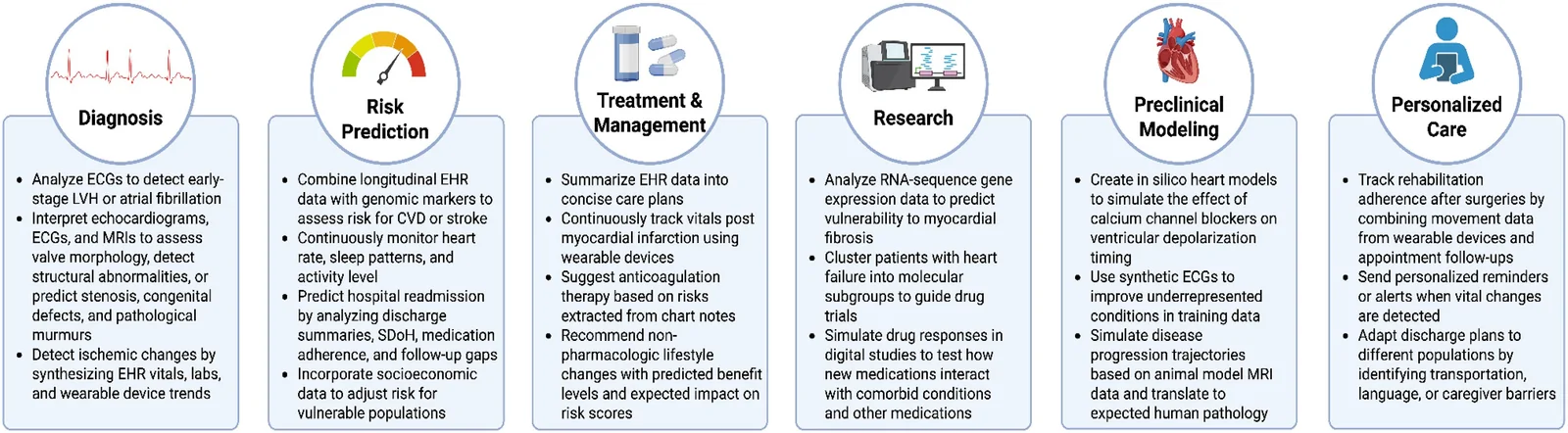
Clinical applications of foundation models across the cardiovascular care continuum.

*Figure 2* illustrates several applications of cardiovascular FMs in supporting both population-level interventions and individual clinical decision-making, including diagnostics, risk prediction, treatment and management, research, preclinical modelling, and patient-centred care. This figure depicts the broad spectrum of clinical applications for cardiovascular FMs spanning both individual patient care and population health management. At the individual level, applications include enhanced diagnostics through early disease detection and accurate classification, risk prediction enabling personalized risk stratification, treatment and management supporting clinical decision-making and therapy optimization, and patient-centred care facilitating continuous monitoring and longitudinal follow-up. At the population and research levels, applications encompass biomarker discovery and drug target identification, preclinical modelling through *in silico* simulations and mechanistic investigations, and public health interventions including resource allocation and preventive strategies. The figure also demonstrates how FMs create an integrated ecosystem supporting the entire cardiovascular care continuum from prevention and early detection through treatment, monitoring, and outcomes research.

## Challenges and limitations

The technical progress summarized above remains at an early stage of clinical translation. Under the evidence-tiering framework introduced in the Methods, only a minority of models discussed in this review meet the Tier 1 standard of evaluation on data independent of their development cohort, and none has yet demonstrated, to the authors’ knowledge, prospective benefit for clinical decision-making, workflow, or patient outcomes. Technical performance metrics should therefore be interpreted alongside the practical barriers to real-world deployment described below.

### Data quality and heterogeneity

Cardiovascular data originate from diverse sources, including ECGs, imaging modalities, genomic sequencing, and wearable devices, each with differing structures and resolutions.^19^ Aligning these modalities during pre-training is technically complex and can introduce error, while heterogeneous patient populations can create feature-distribution shifts that affect model performance.^11,47^ In addition, FMs require extensive data volumes during pre-training, fine-tuning, and validation. Cardiovascular data are often recorded with non-standardized annotations and governed by strict privacy regulations, making high-quality data acquisition difficult and resulting in fragmentation. Advancement in FM applications for cardiovascular care is further hindered by underrepresentation of certain racial groups, women, and individuals from rural or economically disadvantaged communities, leading to biased predictions and generalizability failures.^11^ For example, a systematic review of 486 AI models for CVD risk assessment found that all lacked training data from African individuals and exhibited high risk of bias.^48^ Such underrepresentation in training datasets can lead to systematic inaccuracies in clinical decision-making, particularly affecting underrepresented populations.

### Interpretability and clinical trust

Most FMs function as opaque systems offering limited insight into the processes underlying their predictions, posing significant barriers to clinical adoption.^10^ Physicians, cardiologists, and other specialists require transparent reasoning to trust AI-generated outputs, especially when recommendations impact critical decisions regarding medication adjustments or surgical interventions.^49^ Although tools such as saliency maps and attention visualizations are available, they are often insufficient or overly complex to meet the interpretability requirements essential in medical practice.^11^

### Privacy, ethics, and regulatory concerns

Training FMs requires large amounts of sensitive patient data, raising serious privacy concerns. Importantly, even de-identified data can potentially be re-identified, especially when multimodal sources such as imaging and EHRs are linked.^49^ Foundation models are prone to hallucinations, generating seemingly plausible but erroneous responses that pose dangers in critical applications.^54^ These misrepresentations are especially concerning in CVD care, where unsubstantiated outputs might lead to inappropriate medications, delayed treatment, or unnecessary invasive procedures.^48^ The deployment of FMs also challenges medical accountability. When a model makes a harmful recommendation, liability attribution remains unclear—whether to the physician, institution, or model developer.^49^ Similarly, overreliance on AI could disrupt doctor-patient relationships by displacing human-centred care with impersonal automation.^11^

Regulatory agencies such as the FDA and EMA require transparency, explainability, and demonstration of clinical benefit for approval—requirements that many current FMs cannot meet due to their opaque nature and lack of robust external validation.^10^ Regulation of continuously adapting models remains nascent, presenting evolving challenges for compliance with standardized regulatory frameworks.

### Computational and resource requirements

Both pre-training and fine-tuning of FMs require substantial computational power, storage capacity, and energy consumption. Institutions with limited access to high-performance computing infrastructure are often excluded from both model development and deployment.^19^ Furthermore, the inference phase, even with pre-trained models, can be computationally expensive, particularly for time-critical applications such as real-time ECG analysis or multimodal integration in emergency response systems.^49^

### Implementation and integration barriers

Clinical implementation of FMs is costly, requiring advanced computing infrastructure in addition to substantial investments in staff training, information technology systems, and ongoing maintenance.^47^ Many healthcare organizations, particularly those in resource-limited settings, cannot afford to implement and sustain these technologies. Furthermore, the absence of standardized evaluation metrics for cardiovascular FMs prevents meaningful cross-model comparisons and organizational adoption.^48^ In the absence of standardized benchmarks, healthcare organizations may need to develop local evaluation and governance protocols, further complicating deployment.

## Future directions

### Computational efficiency and accessibility

To address computational limitations, advances in model compression, pruning, and knowledge distillation techniques are needed to reduce memory demands while maintaining performance.^19^ Additionally, edge computing approaches enabling local data processing on wearable devices can facilitate low-latency, privacy-preserving inference for cardiovascular monitoring in rural or ambulatory care settings.^49^

### Multimodal integration and real-time applications

Multimodal models integrating data from diverse sources—including text, ECGs, imaging modalities, genomic information, and wearables—offer more comprehensive assessments of cardiovascular risk and disease.^11^ However, significant technical barriers remain in harmonizing these disparate modalities. Future research should focus on developing architectures that seamlessly integrate multiple data streams while enabling real-time reasoning, particularly for complex conditions such as heart failure or structural heart disease.^47^

Aligning FMs with real-time data from wearable monitoring and remote patient monitoring platforms offers substantial promise. Models trained on ECG data from smartwatches have demonstrated capability in early identification of atrial fibrillation and left ventricular dysfunction.^49^ Integrating these technologies with telehealth platforms could improve cardiovascular risk assessment and enable preventive interventions for populations in rural or under-resourced areas.^11,59,60^

### Standardization and validation

A significant limitation in FM development is the lack of standardized validation protocols. Uncertainty-aware approaches, including conformal prediction and conformalized survival analysis, may complement FM evaluation by quantifying predictive uncertainty rather than reporting discrimination metrics alone.^61^ Future research should emphasize development of external benchmarking datasets and validation frameworks approved by regulatory agencies that evaluate fairness, robustness, and generalizability across diverse populations and healthcare systems.^48^

### Clinical integration and workflow optimization

Successful integration of FMs into clinical workflows requires compatibility with existing EHR systems, development of user-friendly interfaces minimizing workflow disruption, and provision of actionable insights from model outputs.^49^ Future pilot studies and phased implementations should examine operational feasibility, clinician engagement, and impact on clinical outcomes while incorporating multidisciplinary oversight.^47^

Intensive collaboration among clinicians, data scientists, and software developers is necessary to ensure model design is informed by clinical needs.^57^ Approaches involving clinical experts in the selection of evaluation metrics and identification of relevant features can help align model outputs with real-world decision-making.^10^ Establishing mechanisms for post-deployment feedback represents an additional critical step for progressive model refinement.^11^

### Equity and inclusive data collection

Addressing demographic gaps in training datasets is essential for equity in AI-driven cardiovascular care.^48^ Future research should prioritize inclusive data collection across geographic, racial, gender, and socioeconomic dimensions, with mandatory reporting of demographic composition in training and validation datasets.^11,58^

### Enhanced interpretability

Current gaps in interpretability represent a core barrier to building clinical trust and gaining regulatory approval. Ongoing efforts to develop inherently interpretable architectures alongside explanatory tools generating medically grounded explanations are essential for deploying FMs in cardiology.^50^ For example, attention-based models for ECG or imaging tasks can provide visual explanations for predictions, while LLMs trained on clinical documentation can be adapted to generate traceable evidence supporting triage or diagnostic decisions.^49^

## Discussion

Foundation models have demonstrated promising effects on both CVD research and clinical practice. Among the most notable developments are ECG-based FMs such as ECG-GPT and ECGFounder.^36,51^ These models employ transformer architectures for zero-shot arrhythmia detection, waveform generation, and signal classification. They have shown generalizability across different leads, formats, and patient settings, including wearable technologies and ambulatory monitoring systems. ECG-GPT, using generative pre-training methods, enables prompt-driven ECG interpretation and flexible queries about specific rhythm findings or disease states.^51^ These developments mark a transition from rigid classification models towards flexible ECG interpretation systems adaptable to diverse clinical needs.

Imaging-based FMs, including EchoFM and CMR-SSL, are also important for cardiovascular image analysis.^22,24^ EchoFM uses a vision transformer architecture to process echocardiogram videos and has demonstrated effectiveness in left ventricular ejection fraction estimation and chamber segmentation.^24^ CMR-SSL supports cardiac MRI tasks such as chamber segmentation and pathology classification across multiple institutions and imaging protocols.^22^ These models can reduce inter-reader variability and support more standardized image interpretation, although prospective workflow and outcome evaluations remain limited.^47^

Multimodal architectures such as CardioGPT, sEHR-ECG-Text, and EchoCLIP illustrate complementary strategies for integrating EHR data, ECG signals, imaging modalities, and clinical narratives.^25,28,29^ CardioGPT focuses on ECG interpretation generation, sEHR-ECG-Text links ECG representations with structured EHR and clinical text, and EchoCLIP aligns echocardiogram videos with expert reports for retrieval, classification, and language-grounded interpretation tasks.^25,28,29^ These multimodal approaches are particularly relevant in cardiology because clinical reasoning often depends on simultaneous interpretation of signals, imaging findings, longitudinal history, and narrative documentation.

Risk prediction models such as CardioRiskNet and the Apple Heart and Movement FM illustrate distinct approaches to longitudinal cardiovascular risk assessment.^26,30^ CardioRiskNet incorporates explainable modules to identify influential variables driving cardiovascular risk predictions.^26^ The Apple Heart and Movement FM, trained on large-scale wearable biosignal data, demonstrates the scalability of wearable-based representation learning for cardiovascular monitoring, although prospective evidence linking such models to improved outcomes remains limited.^30^

### Critical implementation challenges

Several major obstacles should be addressed before cardiovascular FMs can be integrated into routine care. Many models remain unvalidated in diverse populations and clinical settings. Regulatory frameworks should account for adaptive and multimodal models that may influence clinical decision-making, particularly when models continue learning post-deployment or integrate multiple data types through opaque internal mechanisms.^49^

Equity represents a major concern. Training dataset diversity remains a critical gap, with most FMs underrepresenting key populations, creating both scientific and ethical imperatives for inclusive data collection. Federated learning, as demonstrated by the Federated CT Transformer, offers potential for aggregating data from multiple institutions while preserving patient privacy.^19^

Explainability remains limited in many high-performing systems. Despite feature attribution capabilities in models such as CardioRiskNet, a substantial proportion of FMs remain opaque. Additional work is needed to develop explanation modules that are both reliable and clinically relevant, particularly for practicing clinicians.^10^

Implementation requires investment in infrastructure, workforce training, and interdisciplinary collaboration. Deploying models like EchoFM or CardioGPT in hospitals demands robust computing capacity, EHR integration, and staff capable of maintaining and interpreting these systems. Medical education should incorporate AI literacy, enabling clinicians to critically evaluate model outputs and participate in model oversight. Institutions should foster collaboration among data scientists, cardiologists, engineers, ethicists, and patients to ensure safe, equitable, and effective model deployment.^47^

### Path forward

Foundation models may become important assets in cardiovascular medicine by supporting early diagnosis, continuous risk assessment, and clinical decision-making. Models including ECGFounder, Echo-Vision-FM, CardioGPT, and CardioRiskNet illustrate advances in technical capability and workflow design. However, clinical value will depend on transparent research practices, adaptive regulatory environments, diverse datasets, enhanced usability, prospective evaluation, and operational preparedness.

Despite these promising advances, realizing the full potential of FMs in CVD care requires comprehensive efforts spanning research, policy-making, and clinical implementation. Future research should openly disclose model architectures, training dataset compositions, performance metrics on diverse subgroups, and external validation results.^52^ Transparency is necessary to establish trust, enable reproducibility, and strengthen regulatory processes.^48^ Additionally, datasets should diversify to include underrepresented groups across geographic, racial, gender, and socioeconomic dimensions to prevent bias and achieve equitable outcomes. Foundation models that perpetuate healthcare disparities risk exacerbating existing health inequities rather than reducing them.^11^

Foundation model implementation requires sophisticated partnerships involving computer scientists, cardiologists, ethicists, health system administrators, and patients. Active clinician involvement is needed to identify meaningful use cases, review outputs, and adapt models to operational workflows. Artificial intelligence developers should design systems that are interpretable, responsive to feedback, and adaptable to clinical needs.^10^ FDA and EMA guidelines should evolve to accommodate ML systems appropriately. Ethical frameworks should also evolve to address data ownership, consent for model use, and accountability.^49^

Beyond technical validation, researchers should assess how FMs perform in real-world settings, including measuring impacts on clinical outcomes, provider behaviour, workflow integration, and patient satisfaction. Without this evaluation, even high-performing models may fail to meaningfully improve care.^47^ Healthcare providers require training in AI literacy to understand model outputs, recognize limitations, and maintain clinical oversight.^18^ Organizations should promote environments encouraging ethical innovation while building infrastructure supporting FM deployment, including computational resources and data protection measures.^19^

## Conclusion

Foundation models may substantially influence cardiovascular medicine by supporting earlier disease detection, personalized risk stratification, treatment optimization, and patient-centred care. Text-based, image-based, time-series, and multimodal FMs have shown promising performance across diverse CVD applications, but the strength of evidence varies widely across models and clinical tasks. Major challenges remain, including limited interpretability, demographic biases in training data, regulatory uncertainty, computational demands, and clinical integration barriers. Addressing these challenges requires coordinated action across multiple domains. Inclusive data collection practices should ensure representation of diverse populations. Transparent reporting of model architectures, training methodologies, and validation results is essential for building trust and enabling reproducibility. Regulatory frameworks should account for adaptive and multimodal AI systems. Enhanced interpretability tools are needed to support clinical decision-making and regulatory approval. Interdisciplinary collaboration among clinicians, data scientists, ethicists, and patients is critical for safe and effective deployment. The future contribution of FMs to cardiovascular medicine should be determined through integration with real-world clinical workflows and rigorous evaluation of clinical outcomes. By addressing current limitations through evidence-based approaches and maintaining focus on equity, transparency, and patient safety, FMs may improve cardiovascular care delivery and outcomes across diverse populations.

## Data Availability

All data needed to evaluate the conclusions in the paper are present in the paper.
